# Circuit mechanisms of GPe pauses account for adaptive exploration

**DOI:** 10.21203/rs.3.rs-7117998/v1

**Published:** 2025-08-05

**Authors:** Sang Wan Lee, Minryung Song, Shinwoo Kang, Minsu Abel Yang, Doo-Sup Choi

**Affiliations:** KAIST; Korea Advanced Institute of Science and Technology; Soonchunhyang University; Korea Advanced Institute of Science and Technology; Mayo Clinic College of Medicine and Science

## Abstract

The external globus pallidus (GPe) has traditionally been viewed as a relay nucleus within the basal ganglia (BG), but accumulating evidence indicates a more dynamic role in reinforcement learning (RL). One key characteristic of GPe activity—transient pauses in high-frequency discharge (HFD) neurons—is preserved across species, yet its potential implications in RL remains unclear. Here, we developed a neurophysiologically grounded computational model to investigate the origin and role of GPe pauses in RL. Our model successfully replicated a range of empirical observations, including pause dynamics during learning and cue-related activity modulation. We demonstrated that the GPe-subthalamic nucleus (STN) circuit functions analogously to a denoising autoencoder, modulating baseline excitability in downstream BG circuits and that GPe pauses emerge as circuit-level consequences of strong, convergent inhibition from the GPe to STN. Simulations and in vivo recordings revealed that the activity of GPe-STN projecting neurons increases following sudden environmental changes, promoting adaptive exploration by disrupting action value contrast. Intriguingly, this same configuration impairs performance with extended training, suggesting that habitual behavior may benefit from weakened GPe-to-STN projections. These findings provide a unifying framework for understanding GPe pause dynamics and highlight circuit-level distinctions supporting the balance between flexibility and proficiency in RL.

## Introduction

The external the globus pallidus (GPe) was once regarded as a simple relay nucleus within the basal ganglia (BG), leading reinforcement learning (RL) research to focus primarily on the striatum. However, more recent studies suggest that the GPe plays a broader role in RL^1–7^ through its modulatory influence on BG circuitry^8–10^. Notably, the GPe is anatomically positioned to modulate the baseline excitability of both itself and downstream BG output nuclei—namely, the internal globus pallidus (GPi) and substantia nigra pars reticulata (SNr)—primarily through its inhibitory projections to the subthalamic nucleus (STN)^11^.

Despite these advances, the relationship between physiological activity patterns of the GPe and RL remains poorly understood. A particularly elusive feature is the phenomenon of GPe pauses—brief cessation of spiking in high-frequency discharge (HFD) neurons^11–13^. While these neurons typically exhibit high firing rates (mean: ~ 55Hz)^13^, during pauses lasting a few hundred milliseconds, they temporarily cease firing. This pausing activity is a key distinction between the GPe and GPi^11^. Although both structures receive comparable input patterns, pauses are rare (~6%) in the GPi and SNr, but common (~56%) in the GPe^14^. Since their initial observation in 1971^13^, GPe pauses have been reported across multiple species, including humans^15^, non-human primates^14^, rodents^16^, and songbirds^17^. However, their underlying mechanisms and functional roles in RL remain unclear.

Previous studies indicate a nuanced relationship between GPe pauses and behavior. While GPe pauses occur spontaneously during rest, they exhibit increased frequency during movement (e.g., reaching, grasping, lifting)^13^. Some pauses are temporally aligned with actions, though their timing relationships vary^13,14^. The proportion of frequently pausing GPe HFD neurons decreases as instrumental conditioning progresses^14^, and pause likelihood transiently decreases during the presentation of a rewarding cue^18^. A recent study also reported an association between GPe pauses and exploratory behavior^19^. Despite these diverse observations, no unifying framework coherently explains these diverse findings and their underlying circuit mechanisms.

In this study, we developed a novel BG model grounded in prior neurophysiological data to investigate the mechanisms and behavioral implications of GPe pauses in RL. Our model reproduces experimental results from three distinct studies, offering a unified explanation for seemingly disparate findings. We further show that while the circuit characteristics underlying GPe pauses can facilitate adaptive exploration, they may also interfere with the development of performance proficiency during extended training.

## Results

### A new computational hypothesis for the GPe-STN circuit as a denoising autoencoder

**Fig. 1:**
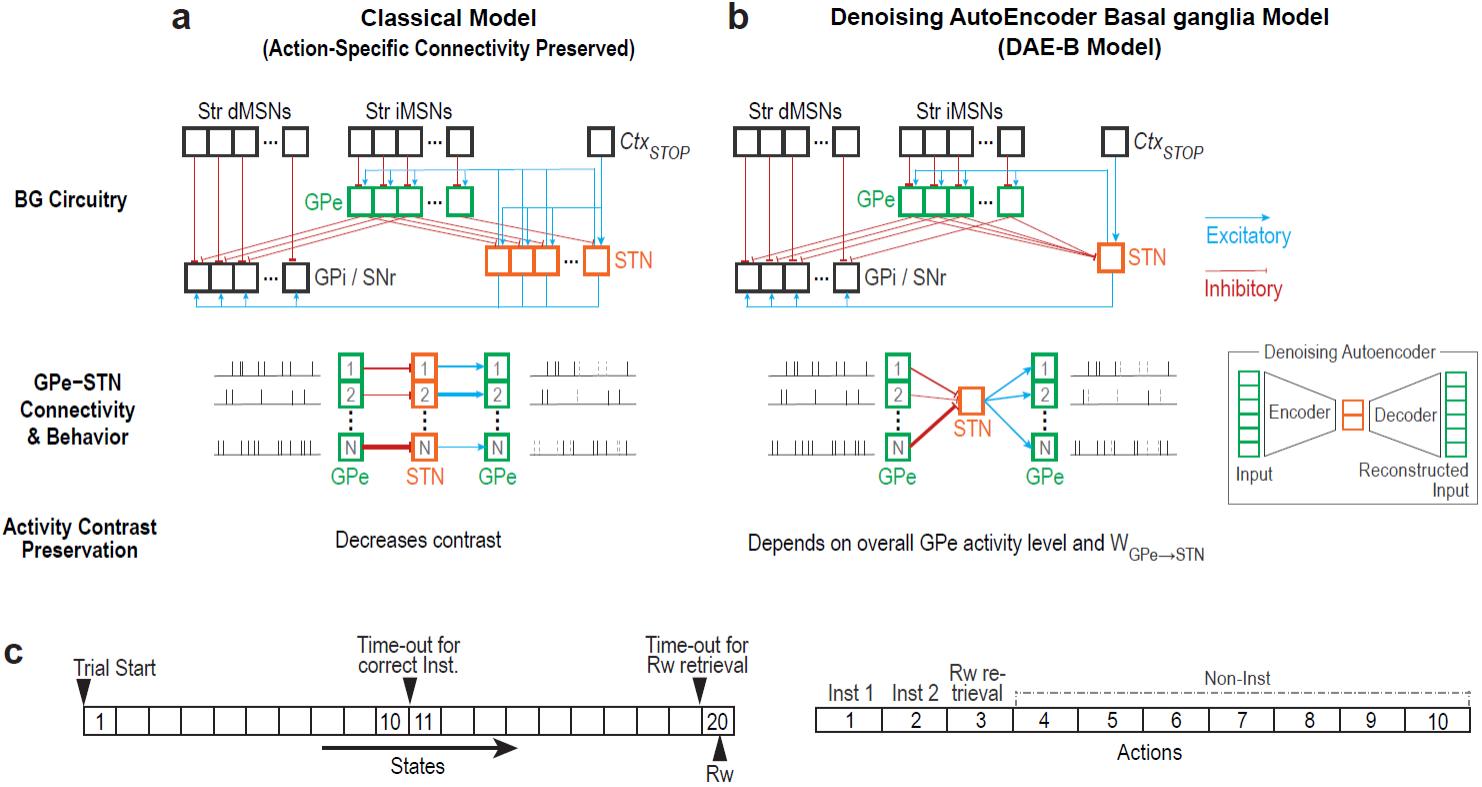
The GPe-STN circuit lacking action-specific connectivity resembles a denoising autoencoder. **a & b**: Structure and behavior of models with (**a**) and without (**b**) action-specific connectivity between the GPe and STN. **Top panels** depict the full BG circuitry. **Bottom panels** illustrate the connectivity pattern and behavior of the GPe-STN circuit. Each box represents a unit in each layer. The numbers inside the boxes in the bottom panels indicate the behavioral option associated with each unit. The inset in **b** shows the structure of a denoising autoencoder. **c**: Task design. **Left** and **right** panels illustrate the states presented to the model and the set of available actions, respectively.

Our model is designed to fully accommodate the canonical basal ganglia circuitry (Fig. 1). Compared to the projections from the striatum to the GPe, the GPe-to-STN connections are more convergent: a single pallidal neuron receives input from less than 0.1% of striatal projection neurons, whereas a single STN neuron receives input from less than 2% of GPe neurons^12,20^. Given this high level of convergence, GPe-to-STN inhibition was modeled as the average activity of STN-projecting GPe neurons. As a result, unlike previous models^4,21^, our implementation does not preserve action-specific connectivity in the GPe-to-STN, STN-to-GPe, and STN-to-GPi pathways (Fig. 1b, top).

This connectivity pattern mirrors the structure of a denoising autoencoder^22,23^ (Fig. 1b inset), in which convergent input integration and divergent feedback promote abstraction and denoising, ensuring robust propagation of essential features such as contextual information. This architectural analogy suggests the key predictions regarding the function of the GPe-STN circuit.

First, the convergent GPe-to-STN projections allow STN neurons to encode the overall activity level of the GPe, thereby modulating the baseline excitability of pallidal neurons (Fig. 1b, bottom). The impact of the overall GPe activity on baseline modulation increases with the strength of $W_{\text{GPe}\to \text{STN}}$. Consequently, when $W_{\text{GPe}\to \text{STN}}$ and/or overall GPe activity are excessively high, the contrast in activity among GPe units associated with different actions can diminish due to a floor effect. This provides a prediction that GPe pauses arise from this floor effect. Furthermore, within the GPe population, neurons with relatively lower activity levels would be more likely to exhibit pauses.

Second, the structural resemblance between denoising autoencoders and the GPe-STN circuit supports the extraction of important features via denoising, thereby preserving high contrast in activity across GPe units. In contrast, models featuring action-specific connectivity between the GPe and STN reduce this contrast, failing to account for the emergence of GPe pauses, which require a large activity difference between pausing and non-pausing neurons (Fig. 1ab, bottom).

To test these predictions, we implemented our model in strict accordance with neurophysiological constraints (see Methods for details). This model, which we term DAE-B (Denoising AutoEncoder-Basal ganglia), was trained to select the correct response—analogous to left- or right- lever presses, or saccadic movements directed toward a reward-predictive target in animal experiments--and then retrieve the reward as quickly as possible, using a discount factor of 0.9 and a cost of −0.2 per behavioral step (Fig. 1c, left). In practice, animals not only perform instrumental actions but also engage in a variety of non-instrumental, exploratory behaviors (Non-Inst), including rearing, roaming, sniffing, and gazing at task-irrelevant stimuli (Fig. 1c, right). To simulate this, the model incorporated three instrumental and seven arbitrary non-instrumental behaviors, which also enhanced its ability to account for animals’ exploratory actions.

### Autoencoder-like computation of the GPe-STN circuit underlies GPe pauses

**Fig. 2:**
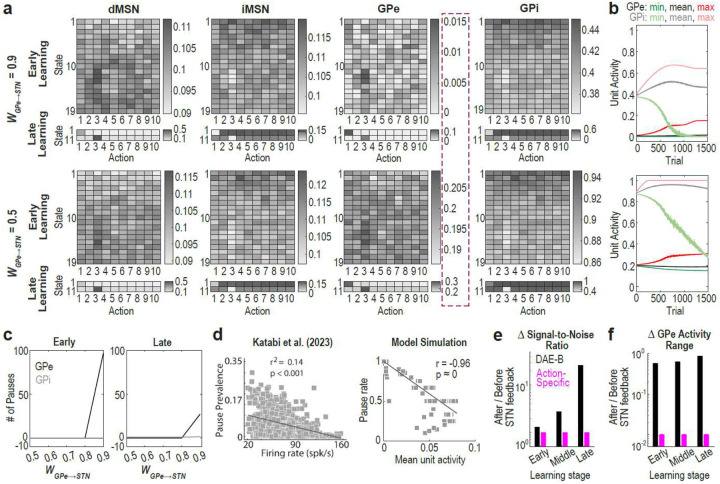
Denoising autoencoder-like GPe-STN circuit dynamics explain pallidal pauses. **a**: Unit activity during early (trial ~100) and late (trial ~1500) stages. Trials of lengths 19 and 2 time steps were selected. **b**: Minimum, mean and maximum activity of GPe and GPi unit activity. **c**: Number of pauses in GPe and GPi during early (trial 100) and late (trial 1500) stages. **d, left**: Correlation between firing rate vs pause prevalence (= pause duration × pause rate) of GPe HFD neurons in animals trained on the task shown in Fig. 2b. Figure adapted from^24^ with permission. **d, right**: Replication of the left panel using model data. r and p indicate the Pearson correlation coefficient and corresponding p-value. Reflecting the fact that animals in Katabi et al. (2023)^24^ were trained for months, simulation results from late stage are shown here; however, significant negative correlations were also found in early and middle stages (see Suppl. Fig. 7a). See Methods for details on the computation of pause rate and mean unit activity. **e & f**: Comparison of models with (pink) and without (black) action-specific connectivity between the GPe and STN. Early, middle and late stages correspond to trial 100, 600 and 1500. **e**: Change in Signal-to-noise ratio (SNR). See Methods for details on the computation of the SNR. **f**: Change in GPe activity range. GPe activity range was defined as the difference between the minimum and maximum activity of GPe units during early, middle, and late learning stages. In **c-f**, $W_{\text{GPe}\to \text{STN}}=0.9$. In **b-f**, data represent results from 100 simulations. In **b**, **c** and **f**, standard error of the means (SEMs) are not visible due to their small size.

To test the first prediction—that GPe pauses arise from a floor effect caused by high overall GPe activity and large $W_{\text{GPe}\to \text{STN}}$—we conducted two analyses: First, we examined whether a large $W_{\text{GPe}\to \text{STN}}$ induces a floor effect on GPe activity. Our findings indicate that when $W_{\text{GPe}\to \text{STN}}$ is large, GPe unit activity is generally low due to weakened excitatory input from the STN (Fig. 2ab) and exhibits frequent pauses (defined as activity level < 1%; Fig. 2c). As $W_{\text{GPe}\to \text{STN}}$ decreases, GPe activity increases and pauses become less frequent. Interestingly, GPi activity was consistently higher than GPe activity (Fig. 2ab), consistent with previous findings (mean firing rate of HFD neurons: 55 Hz in GPe and 63 Hz in GPi in primates)^13^. The significantly higher activity range of GPi compared to GPe may account for why pauses are frequent in the GPe but not in the GPi^13,14^—a key feature of pallidal pauses.

Next, we asked whether GPe units with relatively lower activity levels are more likely to exhibit pauses. Corroborating our hypothesis, a prior study showed that the tendency to pause increases as a neuron’s firing rate decreases^24^. This relationship was reproduced by the DAE-B model (Fig. 2d). This pattern persisted even when unit activity was corrected by calculating mean activity excluding pausing periods (Suppl. Fig. 7b), or when the model was trained on a different task design (Suppl. Fig. 7c).

We then tested the second prediction—that action-specific connectivity disrupts denoising and impairs contrast preservation. We found that the DAE-B model significantly enhanced signal-to-noise ratio (Fig. 2e) and better preserved the range of GPe unit activity following STN feedback (Fig. 2f; Suppl. Fig. 8c). In the model with action-specific connectivity, GPe units with stronger activity received weaker excitatory input from the STN, and vice versa (Fig. 1a bottom). This feedback dampens discriminability among action-encoding GPe neurons, causing pauses to occur in an all-or-none fashion depending on the size of $W_{\text{GPe}\to \text{STN}}$ (Suppl. Fig. 8ab). This contradicts experimental findings demonstrating substantial activity contrasts in GPe neurons during pausing (0 Hz) versus non-pausing (~20–160 Hz) periods^24^.

Taken together, the absence of action-specific connectivity between the GPe and STN, enables the circuit to function analogously to a denoising autoencoder, thereby accounting for GPe pause patterns. This architecture is well-suited to extract essential features (e.g., contextual information) and to modulate GPe baseline activity based on those features, while largely preserving activity contrast across GPe neurons. When $W_{\text{GPe}\to \text{STN}}$ is large, the baseline is lowered, resulting in a strong floor effect and frequent pauses.

### The proposed GPe pause mechanism generalizes to various learning contexts

**Fig. 3:**
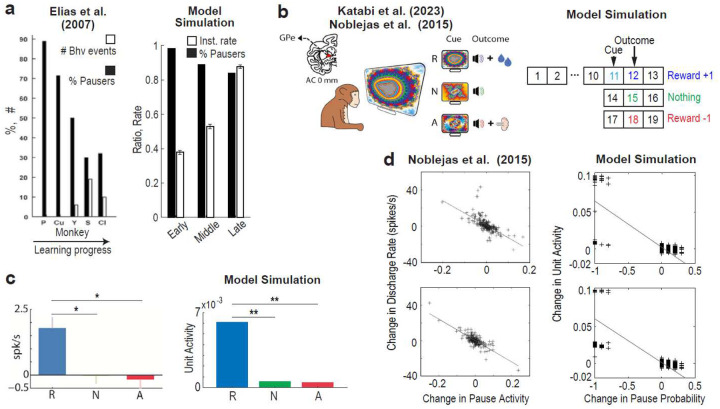
The proposed GPe pause mechanism operates across RL tasks. **a, left**: Figure adapted from^14^. Monkeys S, Cu, Y, and Cl performed self-initiated behavioral tasks. However, most recordings for Monkey Cu were conducted during a “quiet wakeful” state, and Monkey P was not engaged in any task but sat quietly. **a, right**: Replication of the left plot. Pausers were defined as units exhibiting a pause rate > 0.5 per trial. Instrumental response rate was calculated by dividing the total number of Inst1, Inst2 and Reward retrieval (Fig. 1c, right) executions by the trial length. Page’s trend tests revealed a significant increasing trend in instrumental response rates (p = 2.20 × 10^−16^, z = 9.93, L = 1341) and a significant decreasing trend in pauser rates (p = 2.20 × 10^−16^, z = 10.93, L = 1355; tested on reverse order). **b, left**: Experimental design of Katabi et al. (2023)^24^ and Noblejas et al. (2015)^18^. Figure adapted from^24^. **b, right**: Task structure used for model simulation. **c, left**: GPe HFD neuronal responses to cues. Figure adapted from^24^. **c, right**: Replication of the left plot. ****p = 2.56 × 10^−34^ (Wilcoxon rank-sum test). **d, left**: Correlation between changes in discharge rate and pause activity in GPe HFD neurons during cue (top) and outcome (bottom) presentation. Figure adapted from^18^. **d, right**: Replication of the left plot. Pearson correlation analysis yielded: r = −0.82, p = 4.51 × 10^−242^) (top); r = −0.83, p = 3.02 × 10^−251^ (bottom). Pearson correlation analysis restricted to data points with y-values < 0.05 yielded: r = −0.38, p = 3.42 × 10^−35^ (top); r = −0.86, p = 3.21 × 10^−279^ (bottom). **c** and **d, right** show simulation results from the late stage, reflecting the fact that animals in Katabi et al. (2023)^24^ and Noblejas et al. (2015)^18^ were trained for months. **a, c** and **d, right** show results from 100 simulations with $W_{\text{GPe}\to \text{STN}}=0.9$. Error bars in **a** and **c** indicate SEMs but are barely visible due to their small size. Permission for **c** and **d, left** will be obtained upon acceptance.

We then examined whether the DAE-B model could extend to RL contexts by replicating previous experimental findings on GPe neuronal activity and pauses during RL. First, we tested whether the DAE-B model reproduces the inverse relationship between learning progress and the proportion of frequently-pausing GPe neurons, as reported by Elias et al. (2007)^14^. The authors found that, as monkeys became more engaged in instrumental learning tasks—generating more instrumental responses—the proportion of GPe neurons displaying frequent pauses decreased (Fig. 3a left). The DAE-B model successfully reproduced this finding (Fig. 3a right). In the early stages of learning, correct instrumental responses are infrequent, leading to long trial durations, while pauses are widespread (Fig. 2a top; 2c left). As learning progressed, inhibition from indirect pathway medium spiny neurons (iMSNs) to GPe units associated with correct instrumental behaviors decreased, leading to fewer pauses in those GPe units (Fig. 2a top; 2c right). Furthermore, learning enabled the model to execute correct actions more efficiently, shortening trial durations. The combined effect of these changes led to a reduction in frequently pausing units over the course of learning (Fig. 3a right).

Next, we investigated whether the DAE-B model replicates findings from a different task design, in which three distinct cues predicted liquid food, no outcome, and an air-puff, respectively (Fig 3b left)^18,24^. To adapt the task for the model, rewards of 1, 0, and −1 were delivered after the reward, neutral, and aversive cues, respectively (Fig. 3b right). The model had two instrumental behavior options (licking and blinking) and eight non-instrumental behavior options, and was trained to select the appropriate action at the appropriate timing. In the experiment, monkeys tended to lick shortly after the reward cue onset but blinked at the time of air-puff delivery, likely due to the longer latency of the former (Suppl. Fig. 9a). To accommodate this behavioral asymmetry, a half-sized reward was also given when the model selected licking during the reward cue.

Using this RL task, we examined whether the DAE-B model replicates previous experimental findings in two domains: GPe neuronal activity and GPe pausing activity. Katabi et al. (2023)^24^ reported that GPe HFD neurons exhibited significantly higher firing rates during the reward cue compared to neutral or aversive cues (Fig. 3b left). After training with a large $W_{\text{GPe}\to \text{STN}}$, the DAE-B model successfully learned the task (Suppl. Fig. 9b), and replicated this pattern (Fig. 3c right). As the model learned the task, iMSN activity for licking weakened, which was reflected in GPe activity.

We then tested whether the DAE-B model replicates GPe pausing activity during this RL task. Using a similar task design, Noblejas et al. (2015)^18^ demonstrated a negative correlation between changes in firing rates and changes in pause likelihood during both cue presentation (Fig. 3d left top) and outcome delivery (Fig. 3d left bottom). Since a reduction in firing rate increases the phase likelihood (Fig. 2d), this pattern was reproduced in all stages of learning—early, middle, late—in the DAE-B model simulation (Fig. 3d right; Suppl. Fig. 10). With learning, GPe unit activity became increasingly differentiated between correct and incorrect actions, and by the late stage, units associated with correct actions formed distinct clusters in the plot. Notably, the negative correlation remained significant even when units with activity changes greater than 0.05 were excluded.

In sum, the consistency between prior empirical findings and the DAE-B model supports the biological plausibility of the proposed mechanism underlying GPe pauses in RL settings. Our model provides a unified account of disparate findings on GPe pauses within an RL framework.

### GPe-to-STN projection strengthens upon sudden environmental changes

**Fig. 4:**
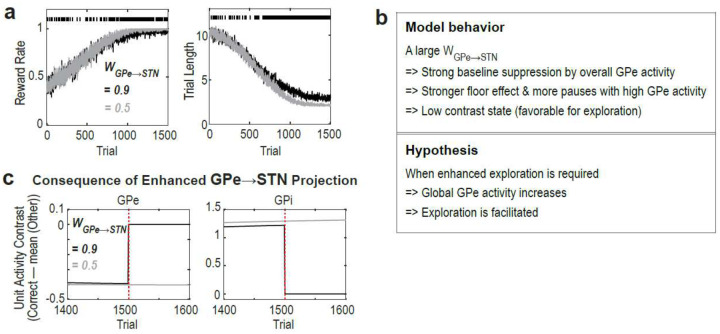
A large $W_{GPe\to STN}$ enables adaptive exploration by modulating GPe-to-STN projections. **a**: Performance of the DAE-B model with $W_{\text{GPe}\to \text{STN}}=0.9$ and 0.5. Upper black squares indicate significant differences (p < 0.05; Wilcoxon rank-sum test). **b**: Schematic summarizing model behavior and the proposed hypothesis. **c**: Unit activity contrast between correct response versus all other actions. GPe-to-STN projections were artificially increased to 1.5 times at trial 1500. Activity was summed across states 1–10. In **a** and **c**, results from 100 simulations were averaged. SEMs are not visible due to their small size.

Since the DAE-B model learned the task slightly better with a small $W_{\text{GPe}\to \text{STN}}$ than with a large one (Fig 4a), we questioned the potential advantage of a large $W_{\text{GPe}\to \text{STN}}$ in RL. In the DAE-B model (Fig. 4b), the global level of GPe activity inhibits baseline GPe activity at the next time step via recurrent GPe–STN interactions, with the strength of this modulation scaling with $W_{\text{GPe}\to \text{STN}}$. Thus, when $W_{\text{GPe}\to \text{STN}}$ is large, a significant rise in overall GPe activity induces a low- contrast state through a strong floor effect. Simulations with the DAE-B model further demonstrated that, under a large $W_{\text{GPe}\to \text{STN}}$, enhancing GPe-to-STN projection by 1.5 times abolished the activity contrast between correct versus incorrect behaviors in both the GPe and GPi (Fig. 4c)—a condition favorable for exploring actions with low values. This effect was not observed when $W_{\text{GPe}\to \text{STN}}$ was small.

These findings led to a new hypothesis: (1) in situations requiring enhanced exploration—such as sudden environmental changes—GPe-to-STN projection strength would increase, and (2) this increase would promote exploration by disrupting established action preferences.

**Fig. 5:**
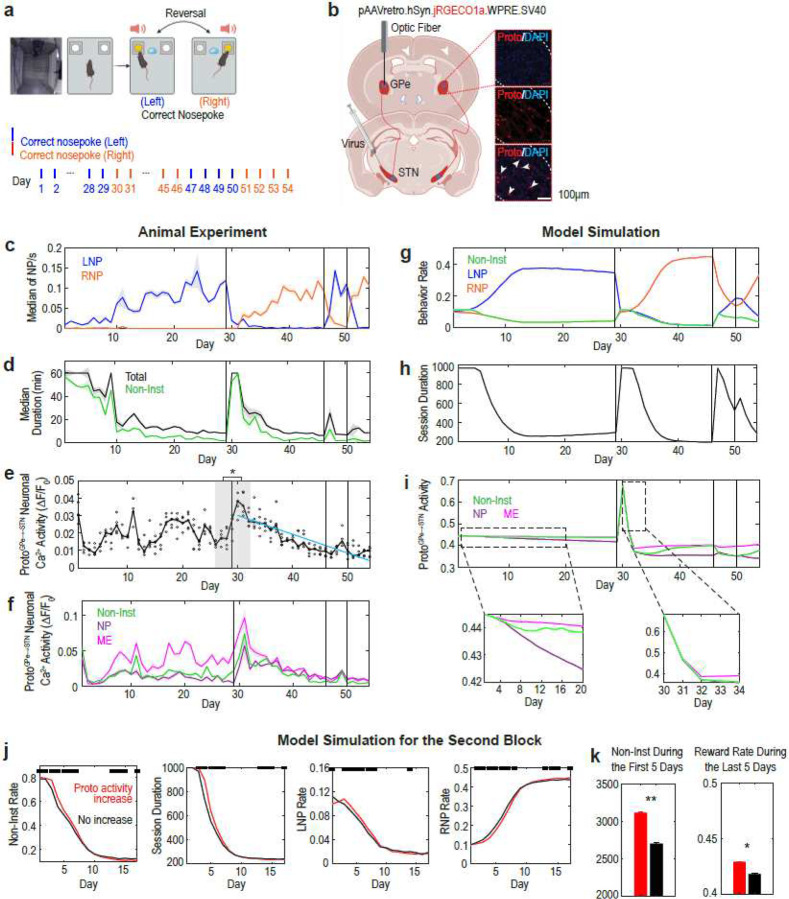
${\text{Proto}}^{GPe\to STN}$ activity increase upon reversal enhances exploration under a large $W_{GPe\to STN}$. **a**: Animal experiment design (top) and reversal schedule (bottom)^25^. **b**: Histological image for ${\text{Proto}}^{\text{GPe}\to \text{STN}}$ neurons. **c**: Rates of instrumental behaviors (N = 5). **d**: Session duration (black) and the time spent on Non-Inst (green). **e**: Calcium activity in ${\text{Proto}}^{\text{GPe}\to \text{STN}}$ neurons. Comparing the mean of the last three days before and the first three days after the reversal (gray shaded area), p = 6.33 × 10^−4^ (paired t-test) and p = 0.0625 (Wilcoxon signed-rank test). **f**: ${\text{Proto}}^{\text{GPe}\to \text{STN}}$ activity during ME, NP and Non-Inst. **g-i**: With $W_{\text{GPe}\to \text{STN}}=0.9$, the model replicates experimental results in **e**-**h**. In **k**, bottom plots are enlargements of the dotted boxes in the top plot. **j**-**k**: Model behavior with (red) and without (black) ${\text{Proto}}^{\text{GPe}\to \text{STN}}$ activity increase following reversal. In **j**, upper black squares indicate significant differences (p < 0.05; Wilcoxon rank-sum test). In **k**, **p = 6.03 × 10^−30^ and *p = 4.23 × 10^−14^ (Wilcoxon rank-sum test). In **g**-**k**, 100 simulation results were averaged and SEMs are not visible due to their small size.

To test the first part of this hypothesis, we conducted an experiment in which mice were trained to nose-poke (NP) the correct hole and enter the magazine (ME) to retrieve a reward (Fig. 5a, top)^25^. After training, the correct NP hole was reversed to the other side (from left to right) without notice and reversed twice more thereafter (Fig. 5a, bottom). The activity of GPe prototypic neurons projecting to the STN (${\text{Proto}}^{\text{GPe}}\to \text{STN}$; Proto from prototypic neurons, which are putative HFD neurons in rodents) was recorded throughout the experiment (Fig. 5b).

Mice successfully learned the task (Fig. 5c), and exploratory, non-instrumental (Non-Inst) behaviors increased following reversals (Fig. 5d). Non-Inst behaviors were defined as any behavior during the session, excluding intervals from −1 to 1 s around NP, the period between magazine entrance and exit, −1 to 0 s before magazine entrance, and 0 to 1 s after magazine exit.

The results confirmed our hypothesis. The ${\text{Proto}}^{\text{GPe}\to \text{STN}}$ activity significantly increased immediately after the first reversal compared to before the reversal (shaded area in Fig. 5e) and then gradually decreased (blue line in Fig. 5e; repeated-measures correlation r = −0.83, p = 5.44×10^−31^). This result is also consistent with previous findings in monkeys^5^, where firing rates of GPe HFD neurons peaked when monkeys had to explore different choices following an unexpected rule change, but gradually decreased over consecutive trials as the newly correct choices became routine.

### Enhancing GPe-to-STN projection promotes exploration

After showing that the GPe-to-STN projections get stronger in response to sudden environmental changes, we next examined the second part of our hypothesis—that enhanced GPe-to-STN projections promote exploration. To simulate the observed increase in ${\text{Proto}}^{\text{GPe}\to \text{STN}}$ activity after reversal, we artificially doubled its activity following the reversal and then set it to gradually decrease (see Methods). Under this implementation, and with a large $W_{\text{GPe}\to \text{STN}}$, relative differences in GPe activity across actions were flattened, resulting in prevalent pauses (Suppl. Fig. 11). The model successfully replicated the experimental results (Fig. 5g–i), including the relative activity difference of ${\text{Proto}}^{\text{GPe}\to \text{STN}}$ during nose-poke (NP), magazine entrance (ME) and Non-Inst behaviors (Fig. 5i).

To evaluate the effect of increased ${\text{Proto}}^{\text{GPe}\to \text{STN}}$ activity on exploration, we compared model behaviors with and without this manipulation (Fig. 5jk). When ${\text{Proto}}^{\text{GPe}\to \text{STN}}$ activity was elevated, exploratory Non-Inst behavior increased following the first reversal. Despite this heightened exploration, task performance also improved: by the end of the second block, previously correct responses (LNP) decreased more, while currently correct responses (RNP) increased more. These results indicate that, when $W_{\text{GPe}\to \text{STN}}$ is large, increased ${\text{Proto}}^{\text{GPe}\to \text{STN}}$ activity facilitates RL by promoting exploration.

Our animal data also support this conclusion. After the second and third reversals—when mice adapted more quickly and showed smaller increases in exploratory Non-Inst behaviors compared to the first reversal (Suppl. Fig. 12)—${\text{Proto}}^{\text{GPe}\to \text{STN}}$ activity did not significantly increase (Fig. 3ef). It is likely that other circuits, such as the hippocampus, contributed to faster adaptation following the second and third reversals, reducing the need for ${\text{Proto}}^{\text{GPe}\to \text{STN}}$ activity modulation^26–29^.

Taken together, our experimental and simulation results suggest that overall ${\text{Proto}}^{\text{GPe}\to \text{STN}}$ activity increases in response to exploratory demands, thereby enabling adaptive exploration when $W_{\text{GPe}\to \text{STN}}$ is large.

### Model provides circuit-level account for habitual vs. goal-directed control

**Fig. 6:**
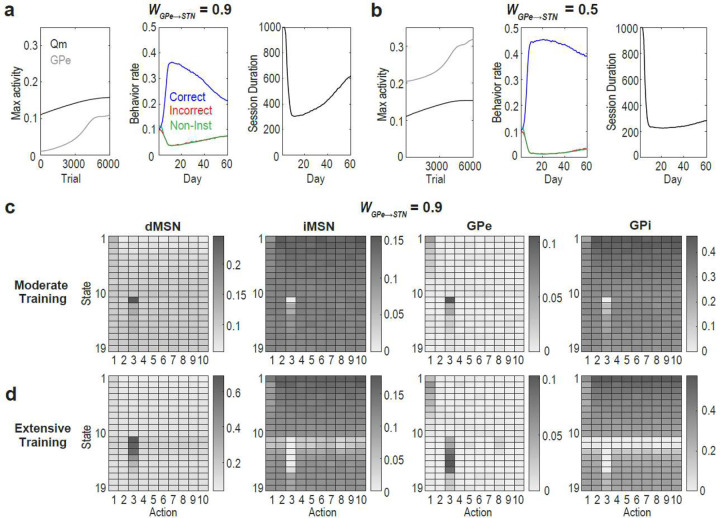
When $W_{GPe\to STN}$ is large, extensive training deteriorates performance. **a & b**: DAE-B Model behavior. Data are averages from 100 simulations. SEMs are not visible due to their small size. **c & d**: Unit activity after moderate (600 trials) and extensive (6000 trials) training.

We also explored the potential disadvantages of a large $W_{\text{GPe}\to \text{STN}}$. We found that when $W_{\text{GPe}\to \text{STN}}$ is large, maximum Qm activity exceeds that of GPe, with the gap increasing with extensive training (Fig. 6a left). This mismatch suggests that learning in Qm cannot be fully accommodated by the GPe, ultimately leading to performance deterioration with extensive training (Fig. 6a middle, right; Fig. 6cd). In contrast, when $W_{\text{GPe}\to \text{STN}}$ is small, maximum Qm activity remained below that of the GPe, and performance deterioration is less pronounced (Fig. 6b). These results suggest that proficiency acquired through extended training (e.g., habit formation) may rely on a distinct circuit characterized by a smaller $W_{\text{GPe}\to \text{STN}}$.

Like the striatum, the GPe and STN are anatomically and functionally divided into limbic, associative, and sensorimotor subregions, with topographic connectivity largely preserved across these territories^30–33^. It is widely accepted that the associative and sensorimotor domains of the BG support goal-directed and habitual behavior, respectively^34–40^. However, it remains unclear why and how the circuit properties of these regions differ to support these distinct roles. The DAE-B model proposes a testable hypothesis: $W_{\text{GPe}\to \text{STN}}$ may be smaller in the sensorimotor territory than in the associative territory. A large $W_{\text{GPe}\to \text{STN}}$ in the associative regions would support adaptive exploration and generate GPe pauses, whereas a smaller $W_{\text{GPe}\to \text{STN}}$ in the sensorimotor territory may facilitate development and maintenance of proficient, habitual performance.

## Discussion

Building on prior neurophysiological findings, we developed the DAE-B model that provides a unified account of the mechanisms underlying GPe pauses and reconciles disparate experimental findings. Our simulation results demonstrate that GPe pauses emerge from the absence of action-specific connectivity between the GPe and STN, combined with a large $W_{\text{GPe}\to \text{STN}}$. Furthermore, using animal experiments and model simulations, we show that this circuit structure enables transient reduction in discriminability among GPe action encodings during adaptive increases in global GPe activity, thereby promoting exploration. We also propose a novel, testable hypothesis that $W_{\text{GPe}\to \text{STN}}$ may be smaller in the sensorimotor territory than in the associative territory in order to support the development and maintenance of proficient performance with extended training. If supported, this hypothesis would suggest a computational basis for the division of labor between associative and sensorimotor domains of the BG.

Our results suggest that the GPe is not merely a relay nucleus within the BG but may function as both a denoising autoencoder and a baseline modulator. Medium spiny neurons in the striatum typically exhibit low firing rates (0.1–10.8 Hz; mean 3.3 Hz)^41,42^. This narrow discharge range constrains both the dynamic range of representable action values and the discriminability among actions. In contrast, pallidal neurons fire at much higher rates, with a broader range (20.3–156.7 Hz; mean 71.1 Hz; somewhat lower in rodents)^24,43^ enabling a wider and more separable action encoding. This distinction necessitates that action values learned in the striatum be amplified in the GPe while suppressing noise. The connectivity pattern between the GPe and STN, which resembles a denoising autoencoder, may support this function while also modulating the baseline excitability of pallidal neurons in a context-dependent manner.

Although we and a previous study^5^ observed that ${\text{Proto}}^{\text{GPe}\to \text{STN}}$ activity increased after sudden environmental changes, the underlying mechanism remains unclear. A prior study reported a correlation between arousal (measured via pupil dilation) and GPe pause occurrences, suggesting that arousal-related regions may be involved^19^. One possibility is enhanced cortical input to the STN during reversal, increasing excitatory drive to GPe neurons^44^. Another possibility is involvement of the corticotropin-releasing factor (CRF) system: CRF neurons in the PVN (Paraventricular nucleus), CeA (central amygdala), and BNST (bed nucleus of the stria terminalis) project to the GPe, where CRFR1 is highly expressed in prototypic but not arkypallidal neurons^45^. CRFR1 activation excites these neurons and, unlike its anxiogenic effects elsewhere, has been shown to promote exploration (e.g., increased distance traveled, speed, and center time)^46,47^. Future studies are warranted to elucidate the mechanisms underlying context-sensitive global modulation of ${\text{Proto}}^{\text{GPe}\to \text{STN}}$ activity. It also remains to be tested whether a general increase in ${\text{Proto}}^{\text{GPe}\to \text{STN}}$ activity actually leads to more pauses.

In addition to prototypic neurons (putative HFD neurons in rodents), the GPe contains arkypallidal neurons (putative low-frequency discharge [LFD] neurons in rodents). These neurons differ markedly from prototypic neurons in both connectivity and activity patterns^48–51^, and they do not typically exhibit pauses^24^. Because our primary interest was the circuit mechanisms underlying GPe pauses, we did not include arkypallidal neurons in the DAE-B model to avoid unnecessary complexity. For similar reasons, we also omitted lateral inhibition among prototypic neurons, as their functional roles and connectivity patterns remain less well characterized and are still under active investigation^48,51–53^. Nevertheless, we cannot exclude the potential contributions of arkypallidal neurons and lateral inhibition to the generation of GPe pauses, and future studies are needed to explore these possibilities.

Previous studies have reported that pauses are not temporally locked to movements^13,14^, whereas our simulation showed pauses coinciding every behavior (Suppl Fig. 13). In the DAE-B model, this occurred because executing any behavior requires stronger suppression of other behaviors, increasing the likelihood of pauses in GPe units associated with those actions. Many experimental and model studies have primarily focused on a limited set of instrumental actions despite the wide diversity of the behavioral repertoire—including saccades^19^—which may have led to an underestimation of pauses that are time-locked to non-instrumental movements. The present study demonstrates that animals spend a substantial amount of time engaged in non-instrumental behaviors. Importantly, a reduction in these behaviors—reflected in the gradual decrease of session duration as learning progresses and the minimal increase in Non-Inst behaviors after the second and third reversals—marks learning progress. Therefore, more closely analyzing non-instrumental behaviors^54^ and incorporating a broad range of non-instrumental behaviors into computational models may deepen our understanding of the exploration-exploitation tradeoff in animals.

Associative and habitual behaviors depend on distinct computations and are mediated by anatomically segregated loops within the basal ganglia (BG)^34,55^. Yet, many computational models treat the BG as a functionally uniform structure and attribute habit formation primarily to Hebbian plasticity in the cortex^56,57^, overlooking extensive evidence implicating the sensorimotor loop^36,37,40,58,59^. Consequently, apart from anatomical connectivity^30,31,33,60^, the circuit-level distinctions supporting domain-specific functions remain poorly understood. The DAE-B model offers a neurophysiological hypothesis that the synaptic strength of GPe-to-STN inhibition differentiates associative and sensorimotor territories. A larger $W_{\text{GPe}\to \text{STN}}$ in associative regions may support flexibility and adaptive exploration via baseline modulation, while a smaller $W_{\text{GPe}\to \text{STN}}$ in sensorimotor regions may stabilize behavior and facilitate habitual performance. This framework links domain-specific computational demands to distinct circuit configurations, offering a mechanistic account of the division of labor across BG subregions.

This study provides a circuit-level framework for understanding the emergence and functional significance of GPe pauses in RL. We show that the GPe–STN circuit, by operating analogously to a denoising autoencoder, enhances exploration in an adaptive manner. Our results call for further investigation into how distinct BG subdomains differentially support behavioral flexibility and proficiency.

## Methods

### DAE-B Model Simulations (Fig. 1–6 except for Fig. 5a–f)

#### Denoising AutoEncoder-Basal ganglia (DAE-B) Model structure

##### STN activity

The STN receives excitatory inputs from cortical areas, while afferents from the GPe constitute its main source of inhibitory input^61^. During sudden needs for rapid action cancellation, additional inputs from the cortex to the STN via the hyperdirect pathway halt behaviors^62,63^. Reflecting these, STN activity was defined as:

(1) $$\text{STN}=B_{\text{STN}}+Ctx_{\text{STOP}}-\text{GPe}-to-\text{STN}\;\text{inhibition}.$$

where $B_{\text{STN}}$, the baseline STN activity, was set to 0.5, and $Ctx_{\text{STOP}}$ (representing cortical stop signal input) was fixed at 0.

At each state, STN activity is initially computed with GPe-to-STNinhibition=0. This preliminary value is used to compute GPe activity (Eq. 3), which in turn is fed into the STN calculation (Eqs. 1–2). The updated STN value is then used to recompute GPe activity (Eq. 3), which is subsequently passed downstream.

##### GPe-to-STN inhibition

The primary excitatory input to both the GPe and GPi is originated from the STN^11^. In turn, GPe prototypic neurons (putative HFD neurons in rodents) send inhibitory projections to the STN (${\text{Proto}}^{\text{GPe}\to \text{STN}}$). Considering the convergent projection patterns from the GPe to the STN^12,20^, GPe-to-STN inhibition was defined as mean activity of ${\text{Proto}}^{\text{GPe}\to \text{STN}}$, given by:

(2) $$\text{GPe}-to-\text{STN}\;\text{inhibition}=\text{mean}\left(\text{STN}-Qm\right)\times W_{\text{GPe}\to \text{STN}},$$

where Qm represents the activity of iMSNs in the striatum and $W_{\text{GPe}\to \text{STN}}$ denotes the synaptic strength from GPe to STN.

##### GPe and GPi/SNr activity

Activity of GPe and GPi/SNr neurons was defined as:

(3) $$\text{GPe}=\text{STN}\times W_{\text{STN}\to \text{GPe}}-Qm,$$

(4) $$\text{GPi}/\text{SNr}=\text{STN}\times W_{\text{STN}\to \text{GPi}}-Qp-\text{GPe}\times W_{\text{GPe}\to \text{GPi}}.$$

Qp represents the activity of dMSNs. To amplify learning in Qm, $W_{\text{GPe}\to \text{GPi}}$ was set to 2, although other values such as 1 or 3 also performed well (Suppl. Fig. 1–4). The hyperdirect pathway enables rapid inhibition of pre-planned movements by transmitting excitatory cortical input directly to the subthalamic nucleus (STN), which then activates inhibitory output nuclei such as the GPi/SNr to suppress motor execution^62,63^. Reflecting this, $W_{\text{STN}\to \text{GPi}}$ was calibrated to cancel all behavioral output when $Ctx_{\text{STOP}}=1$ and fixed at 4 ($W_{\text{STN}\to \text{GPi}}-W_{\text{dMSN}\to \text{GPi}}-W_{\text{GPe}\to \text{GPi}}\geq 1$). All other weights were fixed at 1.

##### Range of $W_{\text{GPe}\to \text{STN}}$

The activity of each unit was bounded within [0, 1]. To ensure that GPe-to-STN inhibition generally remained within this range, the following inequality was required:

(5) $$B_{\text{STN}}\times W_{\text{STN}\to \text{GPe}}>\text{mean}\left(Qm\right),$$

favoring non-small values of $W_{\text{STN}\to \text{GPe}}$. Approximating Qm≈mean(Qm), The GPe activity after STN feedback can be expressed as:

(6) $$\begin{array}{l} \left\{B_{\text{STN}}-\text{mean}\left(B_{\text{STN}}\times W_{\text{STN}\to \text{GPe}}-Qm\right)\times W_{\text{GPe}\to \text{STN}}\right\}\times W_{\text{STN}\to \text{GPe}}-Qm\\ \approx B_{\text{STN}}\times W_{\text{STN}\to \text{GPe}}\times \left(1-W_{\text{STN}\to \text{GPe}}\times W_{\text{GPe}\to \text{STN}}\right)-\text{mean}\left(Qm\right)\times (1-\left.W_{\text{STN}\to \text{GPe}}\times W_{\text{GPe}\to \text{STN}}\right)\\ =\left(1-W_{\text{STN}\to \text{GPe}}\times W_{\text{GPe}\to \text{STN}}\right)\times \left(B_{\text{STN}}\times W_{\text{STN}\to \text{GPe}}-\text{mean}\left(Qm\right)\right). \end{array}$$

To ensure GPe activity remains within bounds and Eq. 5 holds, the following condition must be satisfied:

(7) $$W_{\text{STN}\to \text{GPe}}\times W_{\text{GPe}\to \text{STN}}<1.$$

Furthermore, Eq. 6 implies:

(8) $$0\leq B_{\text{STN}}\times W_{\text{STN}\to \text{GPe}}-\text{mean}\left(Qm\right)\leq {\left(1-W_{\text{STN}\to \text{GPe}}\times W_{\text{GPe}\to \text{STN}}\right)}^{-1}.$$

To allow a wide range for Qm, a large $W_{\text{STN}\to \text{GPe}}$ and a non-negligible value of $W_{\text{STN}\to \text{GPe}}\times W_{\text{GPe}\to \text{STN}}$ are preferred. For simplicity and in consideration of the hyperdirect pathway, $W_{\text{STN}\to \text{GPe}}$ was fixed at 1.1, while $W_{\text{GPe}\to \text{STN}}$ was systematically varied. However, the model displays a similar performance pattern with other values, such as $W_{\text{STN}\to \text{GPe}}=1.5$ and $W_{\text{GPe}\to \text{STN}}=0.66$ (Suppl. Fig. 5–6).

#### DAE-B Model learning

##### Value learning

Qp and Qm were updated to according to the following rules:

(9) $$\begin{array}{l} Qp=Qp+\alpha \times el\times PE\\ Qm=Qm+\alpha \times el\times \left(-PE\right). \end{array}$$

α (learning rate) was fixed at 0.001. The eligibility trace (el) decayed with a factor of 0.9. The prediction error (PE) was defined as:

(10) PE=Reward-Effort+γ×Q(nextstate,nextaction)-Q(state,action).

following the convention of SARSA learning. Q was defined as 1-GPi, simulating thalamic output. Effort (cost of behavior) was fixed at −0.2. The discount factor (γ) was set to 0.9. Qp and Qm units were initialized to ~0.1 to account for the low baseline activity of MSNs^64^. Actions were selected using a softmax function with a temperature of 0.1, except in Fig. 3b–d, which simulated a different task and used a temperature of 0.05.

##### Reversal

After reversal, ${\text{Proto}}^{\text{GPe}\to \text{STN}}$ activity was initially doubled and then gradually decreased according to the following rule:

(11) $$\begin{array}{l} {\text{Proto}}^{\text{GPe}\to \text{STN}}\\ ={\text{Proto}}^{\text{GPe}\to \text{STN}}\times \left\{1+\left(1-{\text{Inc}}_{\text{Decay}}\times t\right)\right\},\;\text{while}\;1-{\text{Inc}}_{\text{Decay}}\times t>0, \end{array}$$

where t is the number of time steps since reversal and ${\text{Inc}}_{\text{Decay}}$ was fixed at 0.0005.

##### Simulating animal experimental data (Fig. 5j–k)

Mimicking our animal experiment (see below), a session was defined as a set of consecutive trials in which either the cumulative length (excluding the final trial) remained below a threshold $t_{\text{Limit}}$ (set to 1000), or the number of rewards obtained was equal to or less than $Rw_{\text{Limit}}$ (fixed at 100). Next session began with the values of Qp and Qm from the end of the last trial of the previous session.

### Animal Experiment (Fig. 5a–f)^25^

#### Animals

All procedures were approved by the Mayo Clinic Institutional Animal Care and Use Committee. GFAP-Cre (JAX #024098) × DIO-GCaMP6s (JAX #028866) bi-transgenic mice (8–10 weeks old; 5 males) were used. Mice were housed under a 12 h light/dark cycle (lights on at 07:00) and food-restricted to 85% of their initial body weight during behavioral experiments.

#### Instrumental task

Behavioral testing was conducted in operant chambers equipped with an active nose port, an inactive port, a magazine, house light, speaker, and cue lights. Each rewarded nose-poke in the active port triggered auditory and visual cues, followed by delivery of a 20% (w/v) sucrose solution via syringe pump. Inactive nose-pokes had no programmed consequences.

Mice were first trained to retrieve 60 rewards from the magazine (days 1–3). This was followed by a fixed-ratio 1 (FR1) schedule with a 60-minute session limit. Sessions ended early if 60 rewards were earned. To facilitate learning, 10 μL of sucrose was placed as bait in the active port during initial FR1 sessions and removed once response latencies stabilized (<2 s across two consecutive sessions).

#### Stereotaxic surgery and virus injection

Under 1.5% isoflurane in oxygen anesthesia, mice were secured in a stereotaxic frame and received bilateral injections (7 × 10^12^ GC/mL; Addgene #100854) of pAAVretro.Syn.NES-jRGECO1a.WPRE.SV40 into the STN at the following coordinates (from dura, relative to bregma): AP −2.14 mm, ML ±1.65 mm, DV −3.90 mm. Experiments were conducted 4–5 weeks post-injection.

#### In vivo calcium imaging (fiber photometry)

Fiber photometry was used to monitor in vivo calcium dynamics. An optic cannula (200/240 μm diameter, 200 μm core) was implanted in the GPe (AP −0.46 mm, ML +2.0 mm, DV −3.0 mm). The cannula was connected to a patch cord delivering 60 μW light at the fiber tip.

Signals were recorded using a multi-wavelength photometry system (v1.2.0.14, Plexon) with time-division multiplexing. Excitation wavelengths included 410 nm (isosbestic control), 465 nm (GCaMP6s), and 560 nm (jRGECO1a). The 410 nm signal was linearly fitted to both 465 nm and 560 nm signals to correct for motion artifacts and bleaching. ΔF/F was calculated as (signal – fitted 410 nm)/fitted 410 nm for both astrocytic and neuronal indicators.

#### Immunofluorescence

Brains were fixed in 4% paraformaldehyde and cryoprotected in 30% sucrose at 4°C. Coronal sections (40 μm) were cut, mounted on gelatin-coated slides, and coverslipped with VECTASHIELD containing DAPI (Vector Laboratories). Confocal images were acquired using an LSM 700 microscope (Zeiss) with 10× and 63× objectives.

#### Statistical analyses

To generate Fig. 2d (right), GPe activity for each of the 10 action options was collected during the trial of interest: 100 for the early stage, 600 for the middle stage, and 1500 for the late stage. The average unit activity across the trial for each action was taken as the mean unit activity. Pause rate for each action was calculated by dividing the number of states where unit activity was less than 0.01 by the trial length (i.e. the total number of states in that trial). Thus, each simulation yielded 10 pairs of pause rate and mean unit activity. We ran 100 simulations for Fig. 2d and each panel in Suppl. Fig. 7.

To compute the signal-to-noise ratio in Fig. 2e, we ran 100 simulations. Signal was defined as the difference between the activity for the correct response and the mean activity for other responses at state 1. This signal was computed for each simulation, and the square of these values was averaged across 100 simulations to obtain the signal power. Noise power was defined as the mean of the variances computed for each of the 10 action options across 100 simulations.

Fig. 5cd show the raw behavioral data without further preprocessing such as per animal normalization. For Fig. 5ef, the average Ca^2+^ activity (ΔF/F_0_) was computed for each session and each animal, and then averaged across animals. In Fig. 3e, the entire session data were used. In Fig. 3f, data were extracted from specific epochs: 0–1 s after NP for NP, 0–1 s after ME for ME, and −2 to −1 s before NP for Non-Inst.

## Supplementary Material

Supplementary Files

This is a list of supplementary files associated with this preprint. Click to download.

- suppl2eiC12.pdf
- suppl1eiC11.pdf
- SupplFigsLegends.pdf
- suppl3eiC31.pdf
- suppl9behav.pdf
- suppl7pausefr.pdf
- suppl5pvf66gpef151.pdf
- suppl4eiC32.pdf
- suppl12explBehav.pdf
- suppl6pvf66gpef152.pdf
- suppl13alwaysPause.pdf
- suppl11quench.pdf
- suppl8actionspecificconnectivity.pdf
- suppl102015.pdf

## Data Availability

Statistical analyses, including t-tests, Wilcoxon signed-rank tests, Mann–Whitney U tests, and repeated-measures correlations, were performed using MATLAB R2023a and R (v4.4.3). All analysis and simulation code will be made available upon publication.

## References

[1] Kang S. Astrocyte activities in the external globus pallidus regulate action-selection strategies in reward-seeking behaviors. Sci Adv 9, (2023). 10.1126/sciadv.adh9239 PMC10275597 37327345

[2] Baker M. External globus pallidus input to the dorsal striatum regulates habitual seeking behavior in male mice. Nat Commun 14, (2023). 10.1038/s41467-023-39545-8 PMC10338526 37438336

[3] Farries M. A., Faust T. W., Mohebi A. & Berke J. D. Selective encoding of reward predictions and prediction errors by globus pallidus subpopulations. Current Biology 33, 4124–4135.e5 (2023). 37703876 10.1016/j.cub.2023.08.042 PMC10591972

[4] Bogacz R., Martin Moraud E., Abdi A., Magill P. J. & Baufreton J. Properties of Neurons in External Globus Pallidus Can Support Optimal Action Selection. PLoS Comput Biol 12, e1005004 (2016). 27389780 10.1371/journal.pcbi.1005004 PMC4936724

[5] Schechtman E., Noblejas M. I., Mizrahi A. D., Dauber O. & Bergman H. Pallidal spiking activity reflects learning dynamics and predicts performance. Proc Natl Acad Sci U S A 113, E6281–E6289 (2016). 27671661 10.1073/pnas.1612392113 PMC5068334

[6] Lilascharoen V. Divergent pallidal pathways underlying distinct Parkinsonian behavioral deficits. Nature Neuroscience 2021 24:4 24, 504–515 (2021). 33723433 10.1038/s41593-021-00810-y PMC8907079

[7] Ging-Jehli N. R. Basal ganglia components have distinct computational roles in decision-making dynamics under conflict and uncertainty. PLoS Biol 23, e3002978 (2025). 39847590 10.1371/journal.pbio.3002978 PMC11756759

[8] Dong J., Hawes S., Wu J., Le W. & Cai H. Connectivity and Functionality of the Globus Pallidus Externa Under Normal Conditions and Parkinson’s Disease. Front Neural Circuits 15, 645287 (2021). 33737869 10.3389/fncir.2021.645287 PMC7960779

[9] Giossi C., Rubin J. E., Gittis A., Verstynen T. & Vich C. Rethinking the external globus pallidus and information flow in cortico-basal ganglia-thalamic circuits. European Journal of Neuroscience 60, 6129–6144 (2024). 38659055 10.1111/ejn.16348

[10] Courtney C. D., Pamukcu A. & Chan C. S. Cell and circuit complexity of the external globus pallidus. Nat Neurosci 26, 1147 (2023). 37336974 10.1038/s41593-023-01368-7 PMC11382492

[11] Jaeger D. & Kita H. Functional connectivity and integrative properties of globus pallidus neurons. Neuroscience 198, 44 (2011). 21835227 10.1016/j.neuroscience.2011.07.050 PMC3221766

[12] Goldberg J. A. & Bergman H. Computational physiology of the neural networks of the primate globus pallidus: function and dysfunction. Neuroscience 198, 171–192 (2011). 21925240 10.1016/j.neuroscience.2011.08.068

[13] DeLong M. R. Activity of pallidal neurons during movement. J Neurophysiol 34, 414–427 (1971). 4997823 10.1152/jn.1971.34.3.414

[14] Elias S. Statistical Properties of Pauses of the High-Frequency Discharge Neurons in the External Segment of the Globus Pallidus. Journal of Neuroscience 27, 2525–2538 (2007). 17344390 10.1523/JNEUROSCI.4156-06.2007 PMC6672489

[15] Vitek J. L. Microelectrode-guided pallidotomy: technical approach and its application in medically intractable Parkinson’s disease. J Neurosurg 88, 1027–1043 (1998). 9609298 10.3171/jns.1998.88.6.1027

[16] Dodson P. D. Distinct Developmental Origins Manifest in the Specialized Encoding of Movement by Adult Neurons of the External Globus Pallidus. Neuron 86, 501–513 (2015). 25843402 10.1016/j.neuron.2015.03.007 PMC4416107

[17] Goldberg J. H., Adler A., Bergman H. & Fee M. S. Singing-Related Neural Activity Distinguishes Two Putative Pallidal Cell Types in the Songbird Basal Ganglia: Comparison to the Primate Internal and External Pallidal Segments. The Journal of Neuroscience 30, 7088 (2010). 20484651 10.1523/JNEUROSCI.0168-10.2010 PMC2874984

[18] Noblejas M. I. Hold your pauses: external globus pallidus neurons respond to behavioural events by decreasing pause activity. Eur J Neurosci 42, 2415–2425 (2015). 26263048 10.1111/ejn.13041

[19] Kaplan A. Spontaneous pauses in firing of external pallidum neurons are associated with exploratory behavior. Communications Biology 2022 5:1 5, 1–6 (2022). 35729350 10.1038/s42003-022-03553-z PMC9213498

[20] Baufreton J. Sparse but selective and potent synaptic transmission from the globus pallidus to the subthalamic nucleus. J Neurophysiol 102, 532–545 (2009). 19458148 10.1152/jn.00305.2009 PMC2712268

[21] Maith O., Baladron J., Einhäuser W. & Hamker F. H. Exploration behavior after reversals is predicted by STN-GPe synaptic plasticity in a basal ganglia model. iScience 26, 106599 (2023). 37250300 10.1016/j.isci.2023.106599 PMC10214406

[22] Vincent P., Larochelle H., Lajoie I., Bengio Y. & Manzagol P.-A. Stacked Denoising Autoencoders: Learning Useful Representations in a Deep Network with a Local Denoising Criterion. The Journal of Machine Learning Research (2010) doi:10.5555/1756006.1953039.

[23] Vincent P., Larochelle H., Bengio Y. & Manzagol P. A. Extracting and composing robust features with denoising autoencoders. Proceedings of the 25th International Conference on Machine Learning 1096–1103 (2008) doi:10.1145/1390156.1390294.

[24] Katabi S., Adler A., Deffains M. & Bergman H. Dichotomous activity and function of neurons with low- and high-frequency discharge in the external globus pallidus of non-human primates. Cell Rep 42, (2023). 10.1016/j.celrep.2022.111898 36596302

[25] Kang S. Pallidal prototypic neuron and astrocyte activities regulate flexible reward-seeking behaviors. bioRxiv 2025.02.10.637554 (2025) doi:10.1101/2025.02.10.637554.

[26] Samborska V., Butler J. L., Walton M. E., Behrens T. E. J. & Akam T. Complementary task representations in hippocampus and prefrontal cortex for generalizing the structure of problems. Nat Neurosci 25, 1314–1326 (2022). 36171429 10.1038/s41593-022-01149-8 PMC9534768

[27] Malá H. Prefrontal cortex and hippocampus in behavioural flexibility and posttraumatic functional recovery: Reversal learning and set-shifting in rats. Brain Res Bull 116, 34–44 (2015). 26033702 10.1016/j.brainresbull.2015.05.006

[28] Vilà-Balló A. Unraveling the role of the hippocampus in reversal learning. Journal of Neuroscience 37, 6686–6697 (2017). 28592695 10.1523/JNEUROSCI.3212-16.2017 PMC6596552

[29] Cernotova D., Stuchlik A. & Svoboda J. Roles of the ventral hippocampus and medial prefrontal cortex in spatial reversal learning and attentional set-shifting. Neurobiol Learn Mem 183, 107477 (2021). 34116140 10.1016/j.nlm.2021.107477

[30] Fraņois C. Behavioural disorders induced by external globus pallidus dysfunction in primates II. Anatomical study. Brain 127, 2055–2070 (2004). 15292054 10.1093/brain/awh239

[31] Karachi C. The pallidosubthalamic projection: An anatomical substrate for nonmotor functions of the subthalamic nucleus in primates. Movement Disorders 20, 172–180 (2005). 15382210 10.1002/mds.20302

[32] Prasad A. A. & Wallén-Mackenzie Å. Architecture of the subthalamic nucleus. Communications Biology 2024 7:1 7, 1–14 (2024). 38200143 10.1038/s42003-023-05691-4 PMC10782020

[33] Alkemade A., Schnitzler A. & Forstmann B. U. Topographic organization of the human and non-human primate subthalamic nucleus. Brain Struct Funct 220, 3075–3086 (2015). 25921975 10.1007/s00429-015-1047-2 PMC4575692

[34] Kim H. F. & Hikosaka O. Parallel basal ganglia circuits for voluntary and automatic behaviour to reach rewards. Brain 138, 1776 (2015). 25981958 10.1093/brain/awv134 PMC4492412

[35] Mizes K. G. C., Lindsey J., Escola G. S. & Ölveczky B. P. Dissociating the contributions of sensorimotor striatum to automatic and visually guided motor sequences. Nat Neurosci 26, 1791–1804 (2023). 37667040 10.1038/s41593-023-01431-3 PMC11187818

[36] Cataldi S. Decreased Dorsomedial Striatum Direct Pathway Neuronal Activity Is Required for Learned Motor Coordination. eNeuro 9, (2022). 10.1523/ENEURO.0169-22.2022 PMC9557335 36171055

[37] Yin H. H., Knowlton B. J. & Balleine B. W. Lesions of dorsolateral striatum preserve outcome expectancy but disrupt habit formation in instrumental learning. European Journal of Neuroscience 19, 181–189 (2004). 14750976 10.1111/j.1460-9568.2004.03095.x

[38] Yin H. H., Knowlton B. J. & Balleine B. W. Blockade of NMDA receptors in the dorsomedial striatum prevents action-outcome learning in instrumental conditioning. Eur J Neurosci 22, 505–512 (2005). 16045503 10.1111/j.1460-9568.2005.04219.x

[39] Shan Q., Ge M., Christie M. J. & Balleine B. W. The Acquisition of Goal-Directed Actions Generates Opposing Plasticity in Direct and Indirect Pathways in Dorsomedial Striatum. Journal of Neuroscience 34, 9196–9201 (2014). 25009253 10.1523/JNEUROSCI.0313-14.2014 PMC6608360

[40] O’Hare J. K. Pathway-Specific Striatal Substrates for Habitual Behavior. Neuron 89, 472–479 (2016). 26804995 10.1016/j.neuron.2015.12.032 PMC4887103

[41] Valsky D. What is the true discharge rate and pattern of the striatal projection neurons in parkinson’s disease and dystonia? Elife 9, 1–27 (2020). 10.7554/eLife.57445 PMC7462612 32812870

[42] Mahon S. Distinct Patterns of Striatal Medium Spiny Neuron Activity during the Natural Sleep–Wake Cycle. Journal of Neuroscience 26, 12587–12595 (2006). 17135420 10.1523/JNEUROSCI.3987-06.2006 PMC6674897

[43] Benhamou L., Bronfeld M., Bar-Gad I. & Cohen D. Globus Pallidus External Segment Neuron Classification in Freely Moving Rats: A Comparison to Primates. PLoS One 7, e45421 (2012). 23028997 10.1371/journal.pone.0045421 PMC3448641

[44] Frank M., Samanta J., Moustafa A. & Sherman S. Hold your horses: impulsivity, deep brain stimulation, and medication in parkinsonism. Science (1979) 318, 1309–1312 (2007). 10.1126/science.1146157 17962524

[45] Hunt A. J. Paraventricular hypothalamic and amygdalar CRF neurons synapse in the external globus pallidus. Brain Struct Funct 223, 2685–2698 (2018). 29569009 10.1007/s00429-018-1652-y PMC5997534

[46] Sztainberg Y., Kuperman Y., Justice N. & Chen A. An Anxiolytic Role for CRF Receptor Type 1 in the Globus Pallidus. Journal of Neuroscience 31, 17416–17424 (2011). 22131403 10.1523/JNEUROSCI.3087-11.2011 PMC6623832

[47] Chang S. Tripartite extended amygdala–basal ganglia CRH circuit drives locomotor activation and avoidance behavior. Sci Adv 8, (2022). 10.1126/sciadv.abo1023 PMC9668302 36383658

[48] Ketzef M. & Silberberg G. Differential Synaptic Input to External Globus Pallidus Neuronal Subpopulations In Vivo. Neuron 109, 516–529.e4 (2021). 33248017 10.1016/j.neuron.2020.11.006

[49] Aristieta A. A Disynaptic Circuit in the Globus Pallidus Controls Locomotion Inhibition. Current Biology 31, 707–721.e7 (2021). 33306949 10.1016/j.cub.2020.11.019

[50] Mallet N. Dichotomous Organization of the External Globus Pallidus. Neuron 74, 1075–1086 (2012). 22726837 10.1016/j.neuron.2012.04.027 PMC3407962

[51] Johansson Y. & Ketzef M. Sensory processing in external globus pallidus neurons. Cell Rep 42, 111952 (2023). 36640317 10.1016/j.celrep.2022.111952

[52] Cui Q. Dissociable Roles of Pallidal Neuron Subtypes in Regulating Motor Patterns. Journal of Neuroscience 41, 4036–4059 (2021). 33731450 10.1523/JNEUROSCI.2210-20.2021 PMC8176746

[53] Jones J. A., Higgs M. H., Olivares E., Peña J. & Wilson C. J. Spontaneous Activity of the Local GABAergic Synaptic Network Causes Irregular Neuronal Firing in the External Globus Pallidus. Journal of Neuroscience 43, 1281–1297 (2023). 36623877 10.1523/JNEUROSCI.1969-22.2023 PMC9987574

[54] Kishi T. Automated analysis of a novel object recognition test in mice using image processing and machine learning. Behavioural Brain Research 476, 115278 (2025). 39357746 10.1016/j.bbr.2024.115278

[55] Balleine B. W. & O’Doherty J. P. Human and rodent homologies in action control: Corticostriatal determinants of goal-directed and habitual action. Neuropsychopharmacology 35, 48–69 (2010). 19776734 10.1038/npp.2009.131 PMC3055420

[56] Frank M. J. Dynamic dopamine modulation in the basal ganglia: a neurocomputational account of cognitive deficits in medicated and nonmedicated Parkinsonism. J Cogn Neurosci 17, 51–72 (2005). 15701239 10.1162/0898929052880093

[57] Topalidou M., Kase D., Boraud T. & Rougier N. P. A Computational Model of Dual Competition between the Basal Ganglia and the Cortex. eNeuro 5, ENEURO.0339–17.2018 (2019). 10.1523/ENEURO.0339-17.2018 PMC6325557 30627653

[58] Turner K. M., Svegborn A., Langguth M., McKenzie C. & Robbins T. W. Opposing Roles of the Dorsolateral and Dorsomedial Striatum in the Acquisition of Skilled Action Sequencing in Rats. Journal of Neuroscience 42, 2039–2051 (2022). 35086903 10.1523/JNEUROSCI.1907-21.2022 PMC8916752

[59] Song M. R. & Lee S. W. Rethinking dopamine-guided action sequence learning. European Journal of Neuroscience vol. 60 3447–3465 Preprint at 10.1111/ejn.16426 (2024). 38798086

[60] Hunnicutt B. J. A comprehensive excitatory input map of the striatum reveals novel functional organization. Elife 5, 1–32 (2016). 10.7554/eLife.19103 PMC5207773 27892854

[61] Hamani C., Saint-Cyr J. A., Fraser J., Kaplitt M. & Lozano A. M. The subthalamic nucleus in the context of movement disorders. Brain 127, 4–20 (2004). 14607789 10.1093/brain/awh029

[62] Schmidt R., Leventhal D. K., Mallet N., Chen F. & Berke J. D. Canceling actions involves a race between basal ganglia pathways. Nat Neurosci 16, 1118 (2013). 23852117 10.1038/nn.3456 PMC3733500

[63] Hannah R. & Aron A. R. Towards real-world generalizability of a circuit for action-stopping. Nat Rev Neurosci 22, 538–552 (2021). 34326532 10.1038/s41583-021-00485-1 PMC8972073

[64] Kim N., Barter J. W., Sukharnikova T. & Yin H. H. Striatal firing rate reflects head movement velocity. Eur J Neurosci 40, 3481–3490 (2014). 25209171 10.1111/ejn.12722

